# Supplementary material for: Cryptic diversity on the genus Caenolestes (Caenolestidae: Paucituberculata) in the Ecuadorian Andes
Source: PeerJ. 2025 Jul 10;13:e19648. doi: 10.7717/peerj.19648 (PMC12256044; doi:10.7717/peerj.19648)
Supplement: Supplemental Information 1 — (A) Nucleotide concatenated (Cytb + RAG1). (B) Amino acids concatenated (Cytb + RAG1). (C) Nucleotide Cytochrome b (D) Amino acid Cytochrome b (E) Nucleotide RAG1 (F) Amino acid RAG1 [file peerj-13-19648-s001.pdf]

Supplemental material 1. Best partitions schemes

A. Nucleotide concatenated (Cyt b + RAG)

Subset	Type	Seqs	Sites	Infor	Invar	Model	Name
1		50	381	71	300	TEST	CytbcodonPos1
2		50	381	11	364	TEST	CytbcodonPos2
3		50	381	286	61	TEST	CytbcodonPos3
4		31	946	12	927	TEST	RagcodonPos1
5		31	946	6	938	TEST	RagcodonPos2
6		31	946	50	873	TEST	RagcodonPos3

Selecting individual models for 6 charsets using BIC...

No.	Model	Score	Charset
1	TIM2e+G4	2892.375	CytbcodonPos1
2	TN+F+I+G4	1446.692	CytbcodonPos2
3	TIM+F+I	8551.971	CytbcodonPos3
4	HKY+F	2942.001	RagcodonPos1
5	HKY+F	2744.759	RagcodonPos2
6	TPM2+F+I	3895.319	RagcodonPos3

B. Amino acids concatenated (Cyt b + RAG)

Subset	Type	Seqs	Sites	Infor	Invar	Model	Name
1		50	381	42	327	TEST	Cytb
2		31	946	18	922	TEST	RAG

Selecting individual models for 2 charsets using BIC...

No.	Model	Score	Charset
1	mtMAM+I	3702.151	Cytb
2	JTTDCMut	6022.232	RAG1

C. Nucleotide Cytochrome b

Subset	Type	Seqs	Sites	Infor	Invar	Model Name
1	50	381	71	300	TEST	codonPos1
2	50	381	11	364	TEST	codonPos2
3	50	381	286	61	TEST	codonPos3

Selecting individual models for 3 charsets using BIC...

No.	Model	Score	Charset
1	TIM2e+G4	2884.510	codonPos1
2	TN+F+I+G4	1449.234	codonPos2
3	TIM+F+I	8493.172	codonPos3

D. Amino acid Cytochrome b

Akaike Information Criterion: mtMAM+I
Corrected Akaike Information Criterion: mtMAM+I
Bayesian Information Criterion: mtMAM+I
Best-fit model: mtMAM+I chosen according to BIC

E. Nucleotide RAG

Subset	Type	Seqs	Sites	Infor	Invar	Model Name
1	31	946	12	927	TEST	Character_Matrix1
2	31	946	6	938	TEST	Character_Matrix2
3	31	946	50	873	TEST	Character_Matrix3

Selecting individual models for 3 charsets using BIC...

No. Model	Score	Charset
1 HKY+F	2931.166	Character_Matrix1
2 HKY+F	2751.631	Character_Matrix2
3 K2P+G4	3778.656	Character_Matrix3

F. Amino acid RAG

Akaike Information Criterion: mtVer+F
Corrected Akaike Information Criterion: mtVer+F
Bayesian Information Criterion: JTT
Best-fit model: JTT chosen according to BIC
