# Supplementary material for: Cryptic diversity on the genus Caenolestes (Caenolestidae: Paucituberculata) in the Ecuadorian Andes
Source: PeerJ. 2025 Jul 10;13:e19648. doi: 10.7717/peerj.19648 (PMC12256044; doi:10.7717/peerj.19648)

Supplemental material 2. **Phylogenetic Trees**

1. Nucleotide concatenated (Cyt b + RAG)

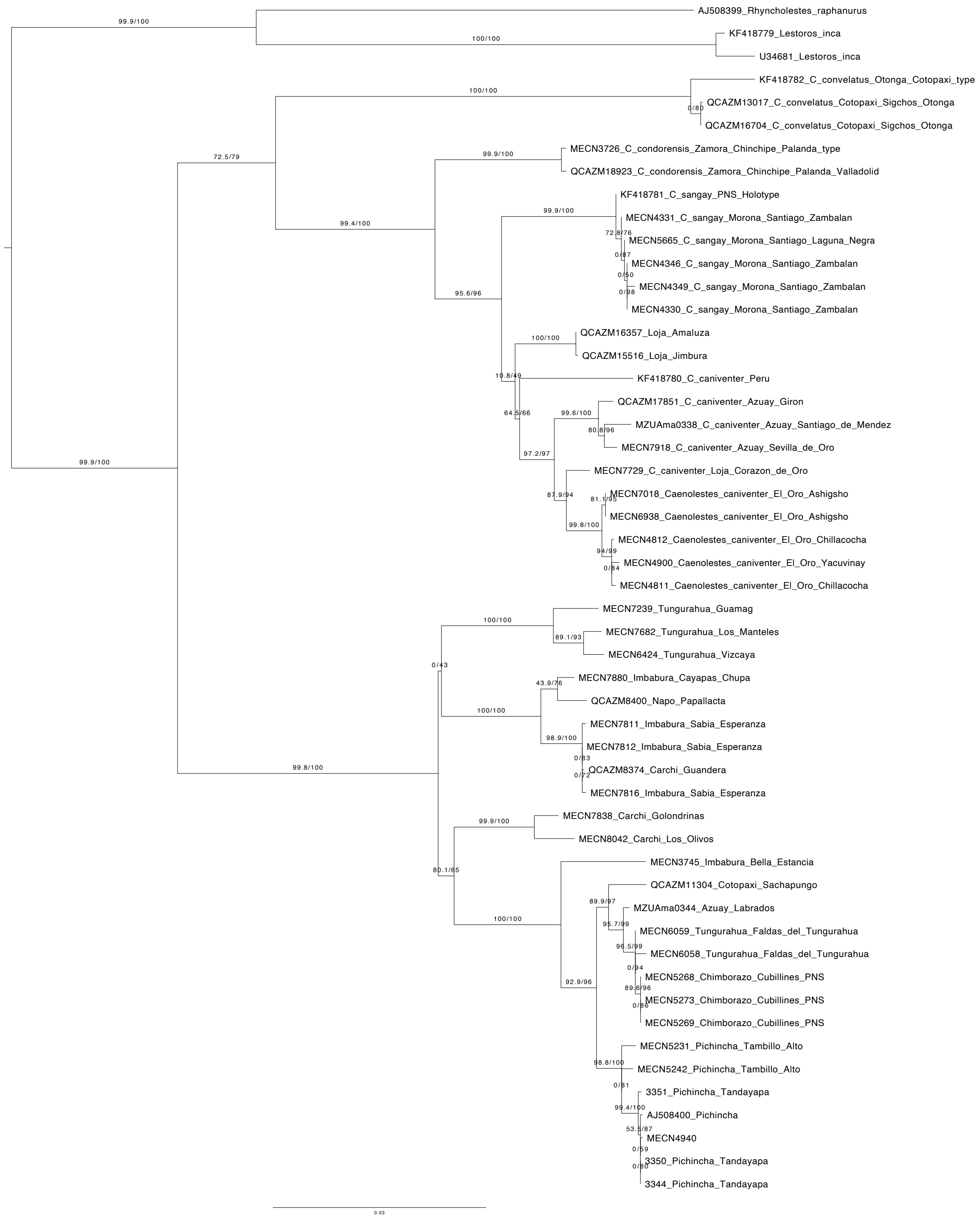

2. Amino acids concatenated (Cyt b + RAG)

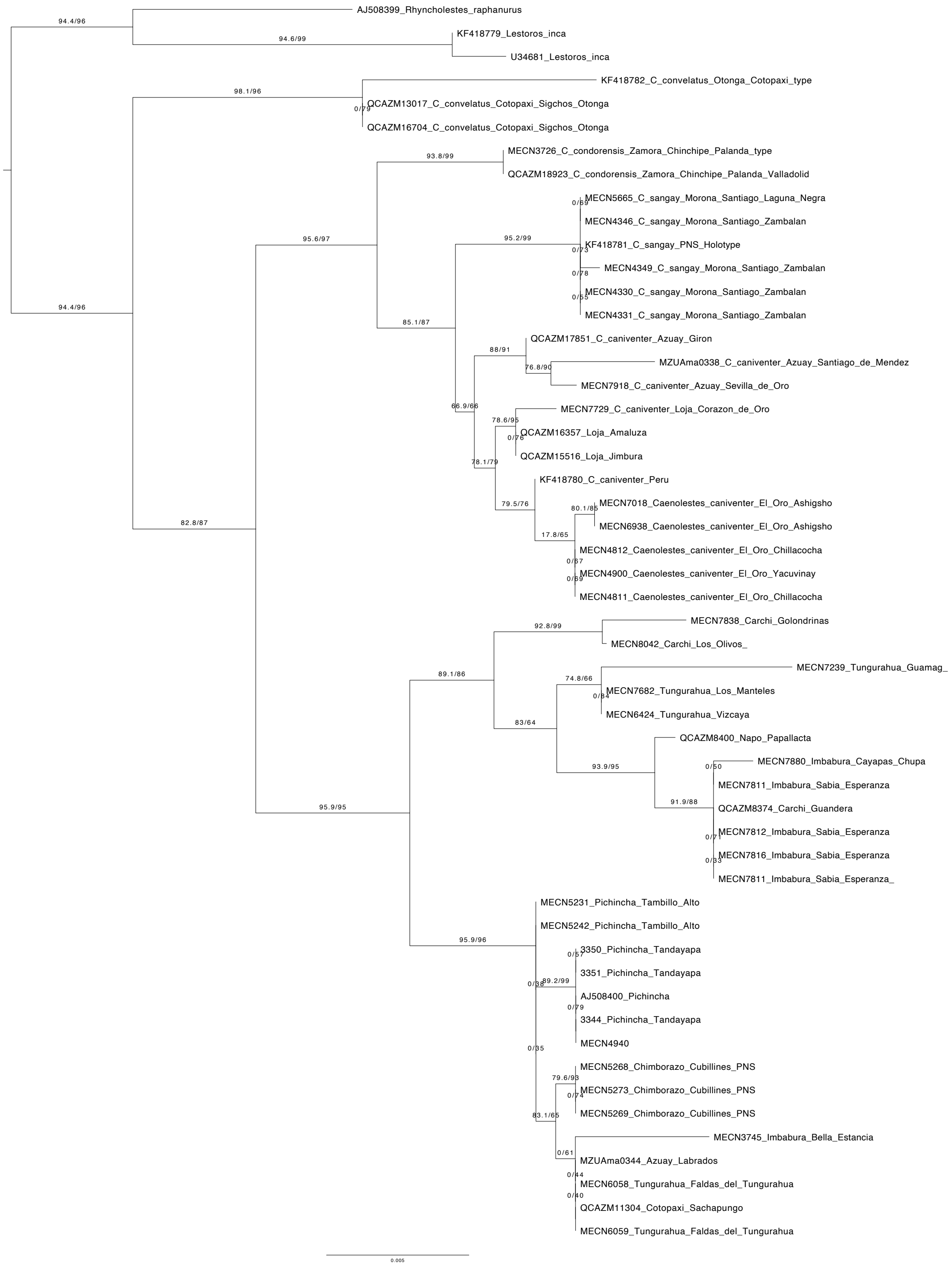

AJ508399_Rhyncholestes_raphanurus
KF418779_Lestoros_inca
U34681_Lestoros_inca
KF418782_C_convelatus_Otonga_Cotopaxi_type
QCAZM13017_C_convelatus_Cotopaxi_Sigchos_Otonga
QCAZM16704_C_convelatus_Cotopaxi_Sigchos_Otonga
MECN3726_C_condorensis_Zamora_Chinchipe_Palanda_type
QCAZM18923_C_condorensis_Zamora_Chinchipe_Palanda_Valladolid
MECN5665_C_sangay_Morona_Santiago_Laguna_Negra
MECN4346_C_sangay_Morona_Santiago_Zambalan
KF418781_C_sangay_PNS_Holotype
MECN4349_C_sangay_Morona_Santiago_Zambalan
MECN4330_C_sangay_Morona_Santiago_Zambalan
MECN4331_C_sangay_Morona_Santiago_Zambalan
QCAZM17851_C_caniventer_Azuay_Giron
MZUAma0338_C_caniventer_Azuay_Santiago_de_Mendez
MECN7918_C_caniventer_Azuay_Sevilla_de_Oro
MECN7729_C_caniventer_Loja_Corazon_de_Oro
QCAZM16357_Loja_Amaluza
QCAZM15516_Loja_Jimbura
KF418780_C_caniventer_Peru
MECN7018_Caenolestes_caniventer_El_Oro_Ashigsho
MECN6938_Caenolestes_caniventer_El_Oro_Ashigsho
MECN4812_Caenolestes_caniventer_El_Oro_Chillacocha
MECN4900_Caenolestes_caniventer_El_Oro_Yacuvinay
MECN4811_Caenolestes_caniventer_El_Oro_Chillacocha
MECN7838_Carchi_Golondrinas
MECN8042_Carchi_Los_Olivos_
MECN7239_Tungurahua_Guamag_
MECN7682_Tungurahua_Los_Manteles
MECN6424_Tungurahua_Vizcaya
QCAZM8400_Napo_Papallacta
MECN7880_Imbabura_Cayapas_Chupa
MECN7811_Imbabura_Sabia_Esperanza
QCAZM8374_Carchi_Guandera
MECN7812_Imbabura_Sabia_Esperanza
MECN7816_Imbabura_Sabia_Esperanza
MECN7811_Imbabura_Sabia_Esperanza_
MECN5231_Pichincha_Tambillo_Alto
MECN5242_Pichincha_Tambillo_Alto
3350_Pichincha_Tandayapa
3351_Pichincha_Tandayapa
AJ508400_Pichincha
3344_Pichincha_Tandayapa
MECN4940
MECN5268_Chimborazo_Cubillines_PNS
MECN5273_Chimborazo_Cubillines_PNS
MECN5269_Chimborazo_Cubillines_PNS
MECN3745_Imbabura_Bella_Estancia
MZUAma0344_Azuay_Labrados
MECN6058_Tungurahua_Faldas_del_Tungurahua
QCAZM11304_Cotopaxi_Sachapungo
MECN6059_Tungurahua_Faldas_del_Tungurahua
94.4/96
94.6/99
98.1/96
0/79
93.8/99
95.6/97
95.2/99
85.1/87
94.4/96
88/91
76.8/90
66.9/66
78.6/95
78.1/79
79.5/76
80.1/85
17.8/65
82.8/87
92.8/99
89.1/86
74.8/66
83/64
93.9/95
91.9/88
95.9/95
95.9/96
89.2/99
79.6/93
83.1/65
0.005

3. Nucleotide Cytochrome b

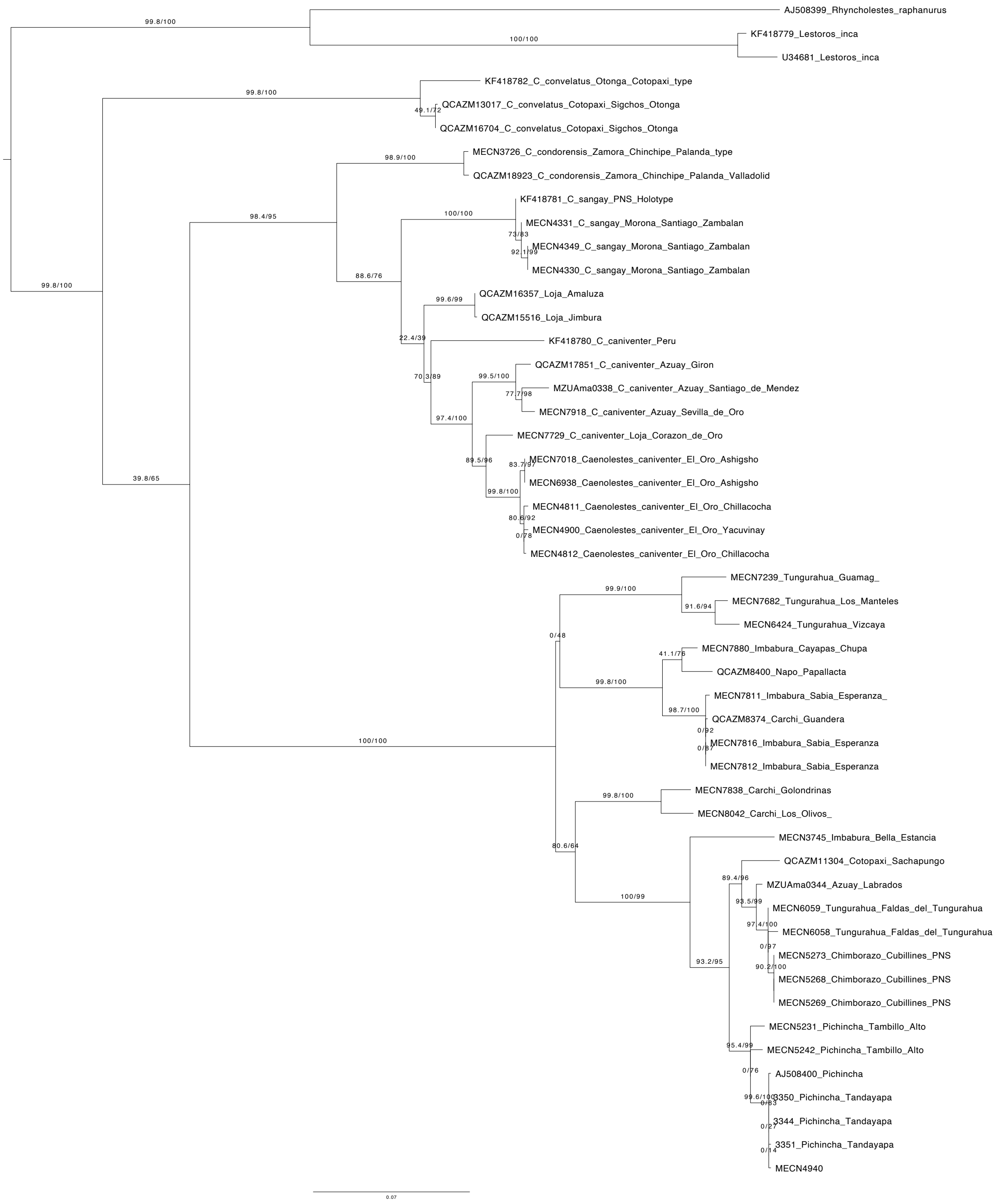

4. Amino acid Cytochrome b

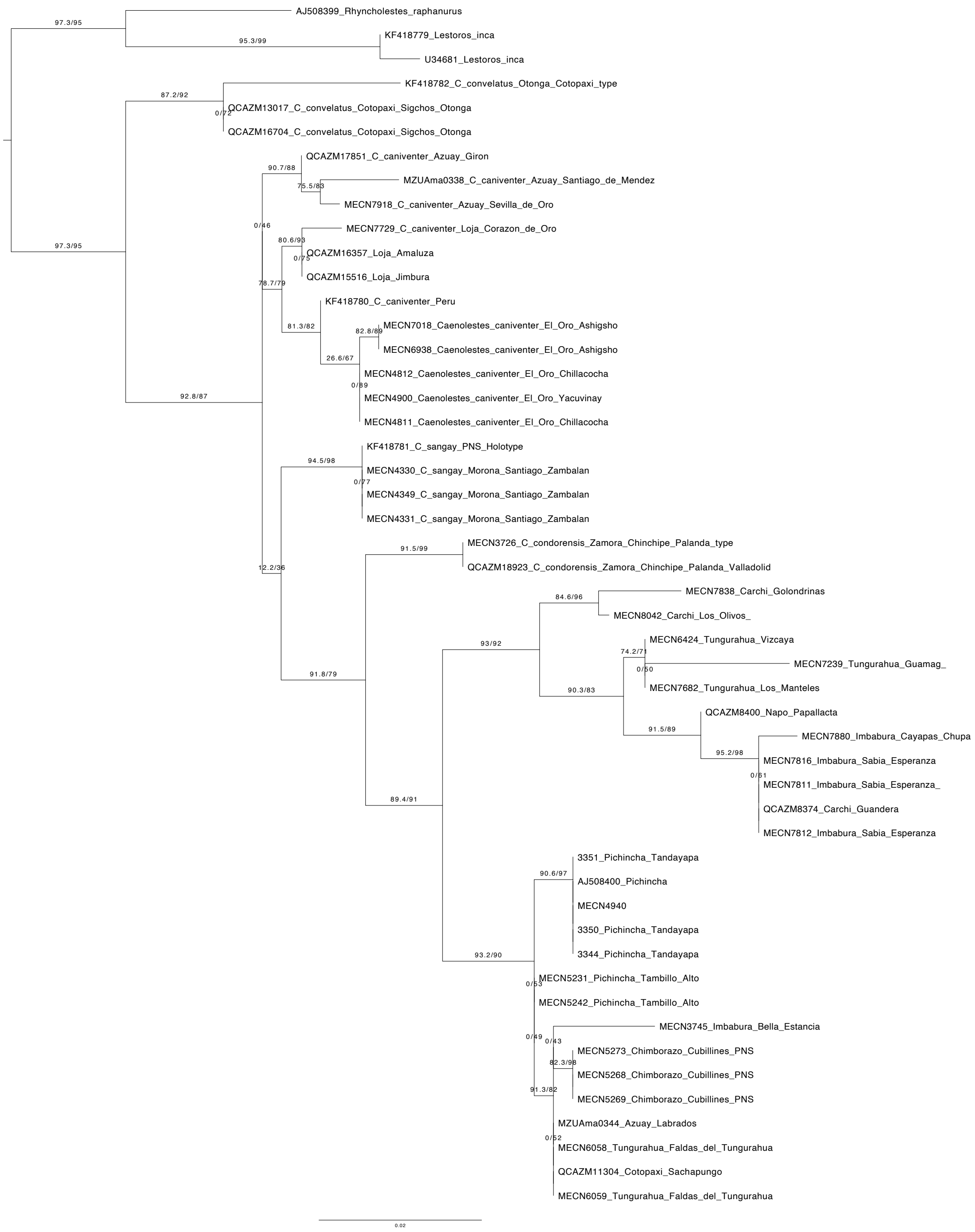

5. Nucleotide RAG

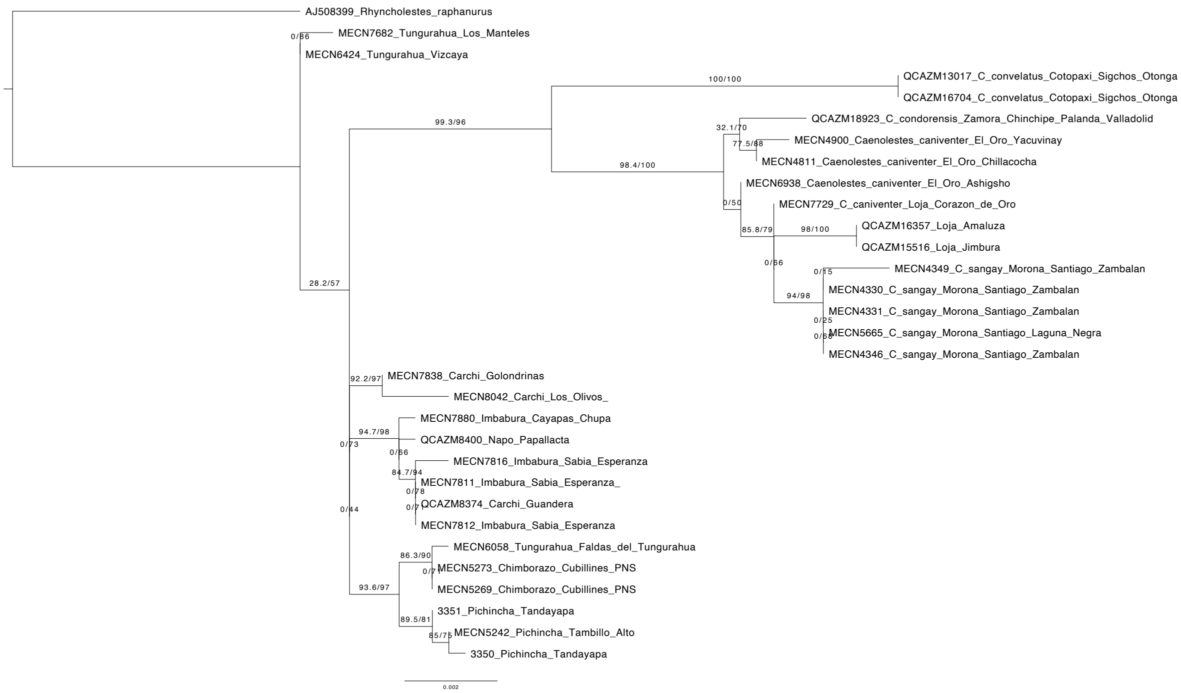

6. Aminoacid RAG

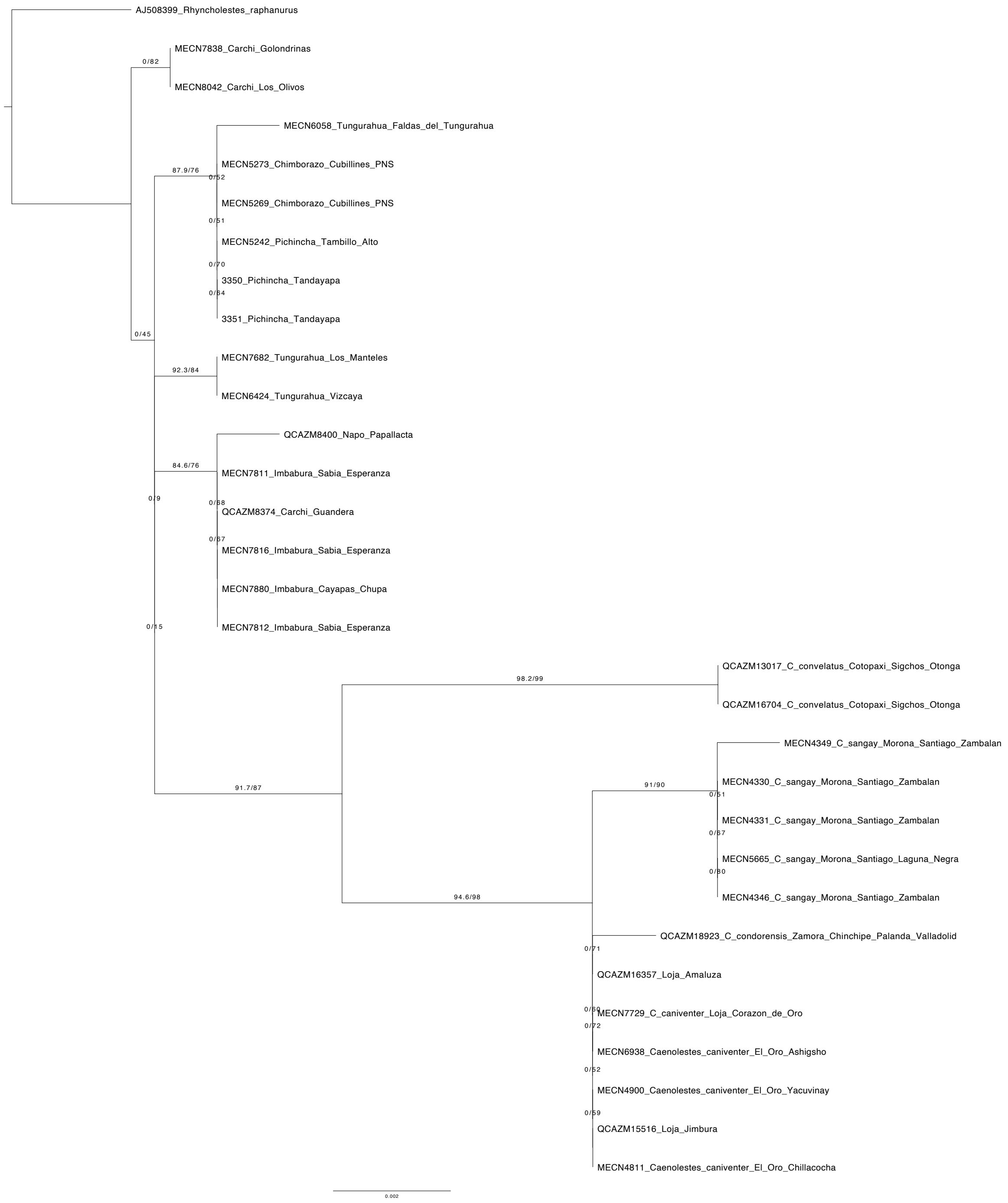

Supplement: Supplemental Information 2 — (A) Nucleotide concatenated (Cytb + RAG1). (B) Amino acids concatenated (Cytb + RAG1). (C) Nucleotide Cytochrome b (D) Amino acid Cytochrome b (E) Nucleotide RAG1 (F) Amino acid RAG1 [file peerj-13-19648-s002.pdf]
