# Supplementary material for: Cryptic diversity on the genus Caenolestes (Caenolestidae: Paucituberculata) in the Ecuadorian Andes
Source: PeerJ. 2025 Jul 10;13:e19648. doi: 10.7717/peerj.19648 (PMC12256044; doi:10.7717/peerj.19648)

Supplemental material 4.

A. Table with tip dates and geographic occurrence data for all samples included in the analysis.

Species	Ocurrence	South American Land Mammal Ages (SALMA)	Age
Stilotherium dissimile	Santa Cruz, Argentina	Late Early Miocene (Santacrucian)	17.5 – 16.3 My
Stilotherium parvum	Gran Barranca, Chubut, Argentina	Early Miocene (Colhuehuapian)	21 – 17.5 My
Gaimanlestes pascuali	Gaiman, Chubut, Argentina.	Early Miocene (Colhuehuapian)	21 – 17.5 My
Pliolestes tripotamicus	Tres Arroyos, Buenos Aires, Argentina	Late Miocene (Huayquerian)	9 – 6.8 My
Pliolestes venetus	Bajo Giuliani, La Pampa, Argentina	Late Miocene (Huayquerian)	9 – 6.8 My
Caenolestoides miocenicus	Gran Barranca, Chubut, Argentina.	Early Miocene (Colhuehuapian)	21 – 17.5 My
Rhyncholestes raphanurus	Valdivian region of Argentina and Chile	Recent	Recent
Lestoros inca	Southern Andes of Peru and Bolivia	Recent	Recent
Caenolestes convelatus	Andes of Colombia and northern Ecuador	Recent	Recent
Caenolestes condorensis	Cordillera del Condor, Ecuador	Recent	Recent
Caenolestes sangay	Western Andes of Ecuador.	Recent	Recent
Caenolestes caniventer	Andes of southern Ecuador and northern Peru	Recent	Recent
Caenolestes fuliginosus	Andes of Colombia and Ecuador	Recent	Recent
Caenolestes sp. 1 Loja	Southeastern of Ecuador	Recent	Recent
Caenolestes sp. 2 Perú	Northern Peru	Recent	Recent
Caenolestes sp. 3 Baños	Central Western Andes of Ecuador	Recent	Recent
Caenolestes sp. 4 Carchi	Northwestern Andes of Ecuador	Recent	Recent
Caenolestes sp. 5 Imbabura	Northeastern Andes of Ecuador	Recent	Recent
Caenolestes sp. 5 Bella Estancia	Northwestern Andes of Ecuador	Recent	Recent

B. List of fossil calibration priors used in MCMCTree, including minimum and maximum node age constraints with corresponding references.

Node	Node maximun age (Ma)	Node minimum age (Ma)	Evidence	Prior
Origin of Caenolestidae	40	30	*Abello et al. 2021*	Uniform
Stilotherium genus	32	28	*Abello et al. 2021*	Uniform
Pliolestes genus	18	16	*Abello et al. 2021*	Uniform
Caenolestoides + extant species split	26	22	*Abello et al. 2021*	Uniform

C. Chronogram showing 95% highest posterior density (HPD) intervals for each node.

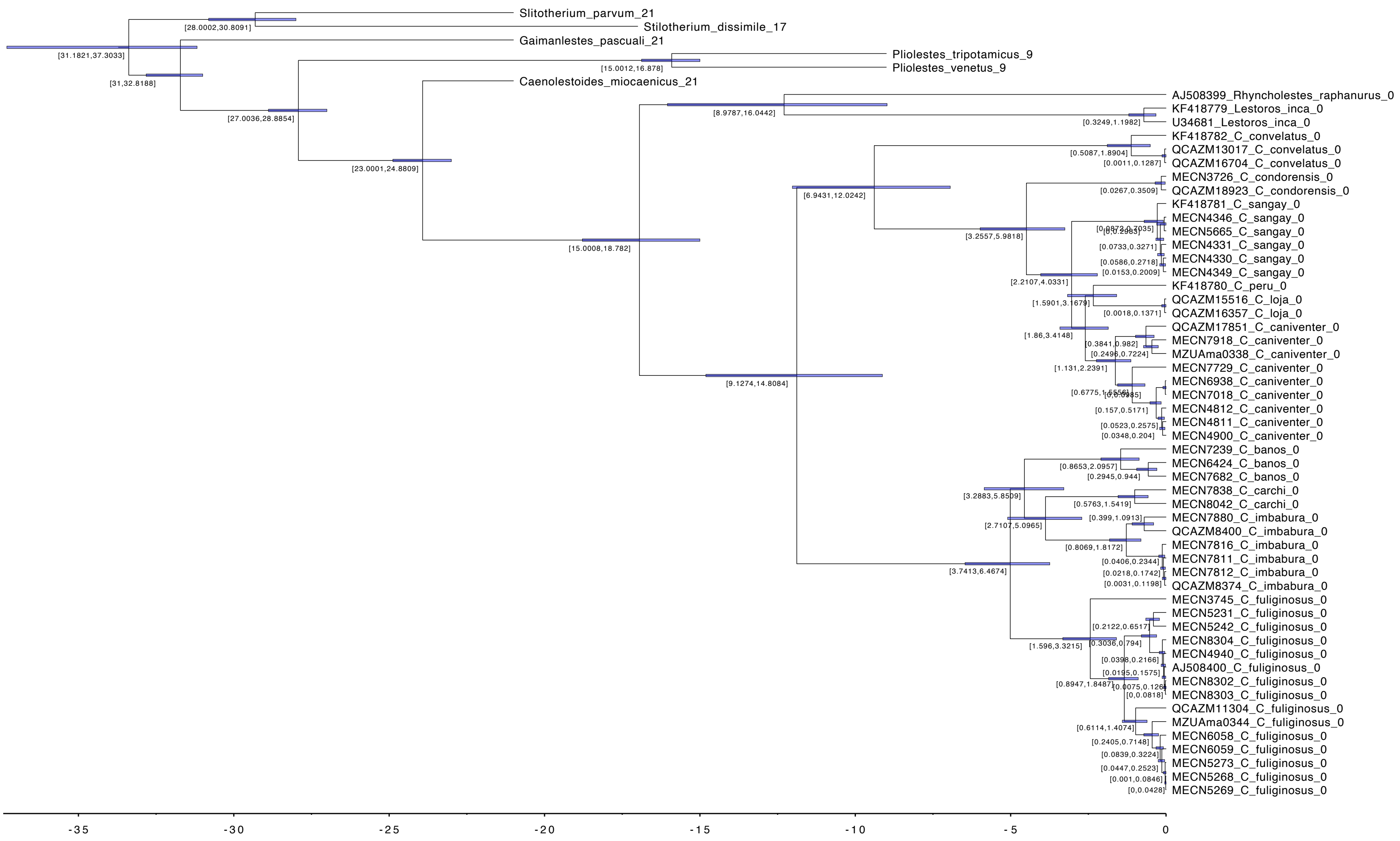

D. Chronogram with estimated mean node ages indicated.

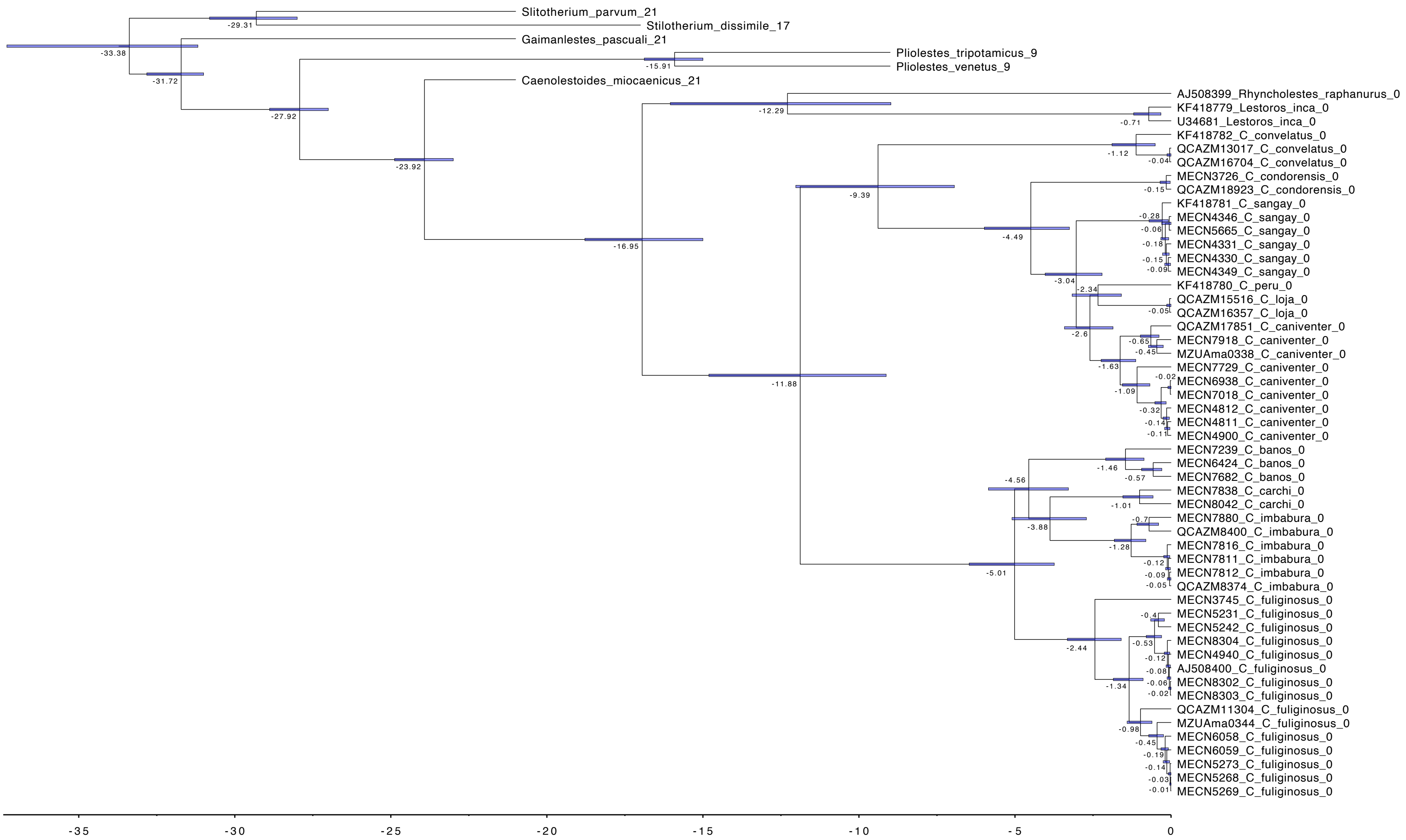

Supplement: Supplemental Information 4 — (A) Table with tip dates and geographic occurrence data for all samples included in the analysis. (B) List of fossil calibration priors used in MCMCTree, including minimum and maximum node age constraints with corresponding references. (C) Dated phylogeny showing 95% highest posterior density (HPD) intervals for each node. (D) Dated phylogeny with estimated mean node ages indicated. [file peerj-13-19648-s004.pdf]
